# Comparison of Glucose, Acetate and Ethanol as Carbon Resource for Production of Poly(3-Hydroxybutyrate) and Other Acetyl-CoA Derivatives

**DOI:** 10.3389/fbioe.2020.00833

**Published:** 2020-07-23

**Authors:** Shenmei Sun, Yamei Ding, Min Liu, Mo Xian, Guang Zhao

**Affiliations:** ^1^CAS Key Laboratory of Biobased Materials, Qingdao Institute of Bioenergy and Bioprocess Technology, Chinese Academy of Sciences, Qingdao, China; ^2^University of Chinese Academy of Sciences, Beijing, China; ^3^Institute of Oceanology, Chinese Academy of Sciences, Qingdao, China; ^4^State Key Lab of Microbial Technology, Shandong University, Qingdao, China

**Keywords:** carbon source, glucose, ethanol, acetate, acetyl-CoA, bioproduction

## Abstract

Poly(3-hydroxybutyrate) (PHB) is a biodegradable and biocompatible thermoplastic, and synthesized from the central metabolite acetyl-CoA. The acetyl-CoA synthesis from glucose presents low atomic economy due to the release of CO_2_ in pyruvate decarboxylation. As ethanol and acetate can be converted into acetyl-CoA directly, they were used as carbon source for PHB production in this study. The reductase mutant AdhE A267T/E568K was introduced into *Escherichia coli* to enable growth on ethanol, and acetate utilization was improved by overexpression of acetyl-CoA synthetase ACS. Comparison of the PHB production using glucose, ethanol or acetate as sole carbon source showed that the production and yield from ethanol was much higher than those from glucose and acetate, and metabolome analysis revealed the differences in metabolism of glucose, ethanol and acetate. Furthermore, other acetyl-CoA derived chemicals including 3-hydroxypropionate and phloroglucinol were produced from those three feedstocks, and similar results were achieved, suggesting that ethanol could be a suitable carbon source for the production of acetyl-CoA derivatives.

## Introduction

**FIGURE 1:**
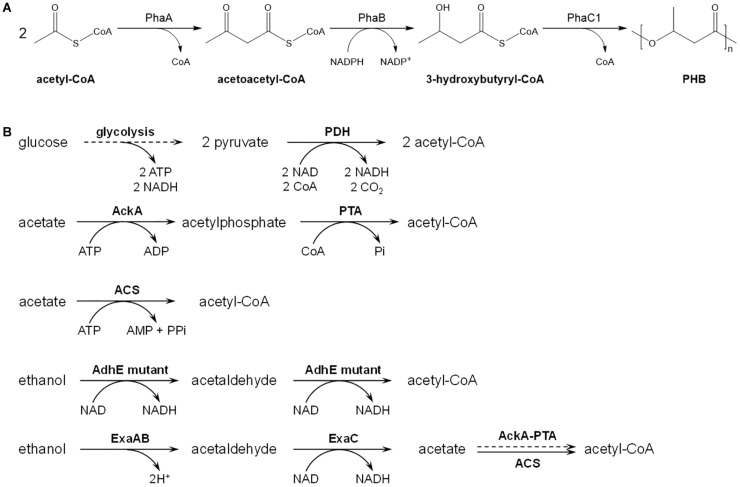
Metabolic pathways for PHB and acetyl-CoA production. **(A)** PHB biosynthetic pathway from acetyl-CoA. PhaA, 3-ketothiolase; PhaB, acetoacetyl-CoA reductase; PhaC1, PHA synthase. **(B)** Different metabolic routes for acetyl-CoA production from glucose, acetate or ethanol. The solid lines represent single step, and dotted line represent multiple steps. The enzymes involved are as follows: PDH, pyruvate dehydrogenase; AckA, acetate kinase; PTA, phsphotransacetylase; ACS, AMP-forming acetyl-CoA synthetase; AdhE, bifunctional acetyl-CoA reductase; ExaA, quinoprotein ethanol dehydrogenase; ExaB, cytochrome c_550_; ExaC, NAD^+^-dependent acetaldehyde dehydrogenase.

Poly(3-hydroxybutyrate) (PHB) was discovered by Lemoigne in 1926 as an intracellular carbon- and energy-storage material, and has attracted much industrial attention as a biodegradable and biocompatible thermoplastic (Chen, 2009). PHB is the most representative homopolymer in polyhydroxyalkanoate (PHA) family, and suitable for applications in packaging, medicine, pharmacy, and food fields. Generally speaking, this polymer is synthesized by polymerization of 3-hydroxybutyryl-CoA catalyzed by PHA synthase PhaC, and 3-hydroxybutyryl-CoA is produced from two acetyl-CoA molecules by β-ketothiolase PhaA and acetoacetyl-CoA reductase PhaB (Figure 1A). In PHB-producing strain *Cupriavidus necator* (formerly *Alcaligenes eutrophus* or *Ralstonia eutropha*), genes encoding these three enzymes are arranged as a cluster *phaCAB* (Steinbuchel and Schlegel, 1991), which has been cloned and expression in *Escherichia coli* leading to successful PHB production in the engineered *E. coli* strains (Zhang et al., 2015; Wu et al., 2016a, b).

Acetyl-CoA is a fundamental metabolite in central metabolic pathways of *E. coli*, and also a precursor for biosynthesis of large number of industrial chemicals and natural products including PHB (Martin et al., 2003; Meng et al., 2012; Liu et al., 2016). The most common metabolic route for acetyl-CoA synthesis in *E. coli* is the glycolysis pathway coupled with decarboxylation of pyruvate by pyruvate dehydrogenase (Bates et al., 1977). Through this pathway, each mol of glucose is converted into 2 mol of acetyl-CoA with generation of 4 mol of NADH, 2 mol of ATP and 2 mol of CO_2_ (Figure 1B). The release of CO_2_ lowers the atomic economy of targeted chemical biosynthetic pathway, leading to decrease of theoretical production yield, titer and productivity (Chae et al., 2017).

Acetate and ethanol are both ordinary and inexpensive commodity chemicals, which can be produced either biologically or chemically. Acetate is manufactured mainly from methanol and carbon monoxide in the presence of rhodium-based catalyst except that a small section used in food industry is produced by fermentation (Zhou and Qian, 2010). Besides fermentation of lignocellulosic biomass (Sun and Cheng, 2002), ethanol can also be produced from coal by chemical methods. There are three ethanol-producing routes from coal with syngas (Pan et al., 2007), acetate (McGinnis, 1983), and dimethyl ether (Liu et al., 2013) as intermediate, respectively, which were all applied in industrial scale. The capacity of ethanol production from coal has exceeded 2 million tons per year in China. Acetate and ethanol can be directly converted into acetyl-CoA by microorganism, and could be considered as alternative substrates for production of acetyl-CoA with high atomic economy, and then production of acetyl-CoA derived chemicals. In *E. coli*, acetyl-CoA is produced from acetate *via* two different pathways, AMP-forming acetyl-CoA synthetase (ACS) catalyzing the higher-efficient pathway and phsphotransacetylase/acetate kinase (Pta-AckA) catalyzing the lower-efficient pathway (Wolfe, 2005). In both pathways, ATP is consumed for the production of acetyl-CoA from acetate, and no reducing power is produced during this process (Figure 1B), indicating that extra acetate is demanded to generate ATP and reducing power when acetate is used as sole carbon source. Previously, we have accomplished the production of phloroglucinol (PG) and mevalonate from acetate in recombinant *E. coli* with ACS overexpression (Xu et al., 2017, 2018).

*E. coli* possesses a bifunctional reductase AdhE, which catalyzes the two-step reduction of acetyl-CoA to acetaldehyde and then to ethanol during anaerobic growth (Kessler et al., 1991). Although both reactions catalyzed by AdhE are reversible, *E. coli* cannot grow aerobically on ethanol as the sole carbon source due to the insufficiency of *adhE* expression (Chen and Lin, 1991) and the susceptibility of AdhE protein to metal-catalyzed oxidation (Tamarit et al., 1998). Further study revealed an AdhE A267T/E568K mutant with acquired and improved aerobic growth ability on ethanol when overexpressed in *E. coli* (Membrillo-Hernandez et al., 2000), suggesting that this AdhE mutant can convert ethanol into acetyl-CoA efficiently and aerobically. *Pseudomonas aeruginosa* ATCC 17933 grows aerobically with ethanol as sole carbon source, and encodes an unusual quinoprotein-dependent ethanol oxidizing system ExaABC, which catalyzes the oxidation of ethanol to acetate (Figure 1B; Diehl et al., 1998; Schobert and Gorisch, 1999). Although the conversion of ethanol to acetyl-CoA through both routes present a carbon recovery of 100%, the pathway employing AdhE A267T/E568K mutant produces more NADH, and does not require energy supply.

To produce PHB from ethanol and acetate, we constructed an engineered *E. coli* strain growing with ethanol as sole carbon source by introduction of AdhE A267T/E568K mutant, and improved the acetate utilization by overexpression of ACS. Comparison of the PHB production using glucose, ethanol or acetate as sole carbon source showed that the production and yield from ethanol was much higher than those from glucose and acetate. Furthermore, other acetyl-CoA derived chemicals including 3-hydroxypropionate (3HP) and PG were produced from those three feedstocks, suggesting that ethanol is a suitable carbon source for the production of acetyl-CoA derivatives.

## Materials and Methods

### Bacterial Strains and Plasmids Construction

All bacterial strains, plasmids and primers used in this study were listed in Table 1. The plasmids pACYCDuet-*adhE* and pACYCDuet-*acs* were constructed by inserting the *adhE* gene (accession No. AP_001865.1) and the *acs* gene (accession No. AP_004570.1) from *E. coli* into pACYCDuet1, respectively. The plasmid pACYCDuet-*adhE*_mut_ was constructed using Fast Site-Directed Mutagenesis Kit (TIANGEN). The *dnaKJ* gene from *E. coli* and POS5 gene from *Saccharomyces cerevisiae* were amplified and inserted between the *Not*I and *Nde*I sites of the plasmid pACYCDuet-*adhE*_mut_ to generate pACYCDuet-*adhE*_mut_-dnaKJ and pACYCDuet-*adhE*_mut_-POS5, respectively.

**TABLE 1:** Bacterial strains, plasmids, and primers used in this study.

**Plasmids, strains and primers**	**Description**	**Source**
**Plasmids**		
pACYCDuet1	T7 promoter, *lacI*^q^, p15A ori, Cm^r^	Novagen
pETDuet1	T7 promoter, *lacI*^q^, pBR322 ori, Amp^r^	Novagen
pET-28a	T7 promoter, *lacI*^q^, pBR322 ori, Kan^r^	Novagen
pACYCDuet-*adhE*	*lac*1-6 promoter, *adhE*, *lacI*^q^, p15A ori, Cm^r^	This study
pACYCDuet-*adhE*_mut_	*lac*1-6 promoter, *adhE* A267T/E568K, *lacI*^q^, p15A ori, Cm^r^	This study
pACYCDuet-*adhE*_mut_-*dnaKJ*	*lac*1-6 promoter, *adhE* A267T/E568K, *dnaKJ*, *lacI*^q^, p15A ori, Cm^r^	This study
pACYCDuet-*adhE*_mut_-POS5	*lac*1-6 promoter, *adhE* A267T/E568K, POS5, *lacI*^q^, p15A ori, Cm^r^	This study
pACYCDuet-*acs*	*lac*1-6 promoter, *acs*, *lacI*^q^, p15A ori, Cm^r^	This study
pBAD-Ae-pha	*araBAD* promoter, *phaCAB*, Amp^r^, *araC*, pBR322 ori	(Aldor and Keasling, 2001)
pET-MCRCN	pBR322 ori, *lacI*^q^, T7 promoter, *mcr*_550__–__1219_ N940V/K1106W/S1114R, *lac*_2–51_ promoter, *mcr*_1__–__549_, Amp^r^	Lab collection
pET28a-*phlD*	*trc* promoter, *phlD*, *lacI*^q^, pBR322 ori, Kan^r^	Lab collection
**Strains**		
*E. coli* DH5α	F^–^, *supE*44, Δ*lacU*169, (*φ*80 *lacZ*Δ*M15*), *hsdR*17, *recA*1, *endA*1, *gyrA*96, *thi*-1, *relA*1	Lab collection
*E. coli* W3110	F^–^, λ^–^, *IN*(*rrnD-rrnE*)1, *rph*-1	Lab collection
*E. coli* MG1655 (DE3)	F^–^, λ(DE3), *ilvG*^–^, *rfb*-50, *rph*-1	Lab collection
*E. coli* BL21(DE3)	F^–^, *ompT*, *gal*, *dcm*, *lon*, *hsdSB*, (rB^–^, mB^–^), λ(DE3)	Lab collection
*S. cerevisiae* CEN.PK2-1C	*MAT*a; *ura*3-52; *trp*1-289; *leu*2-3,112; *his*3Δ1; *MAL*2-8^C^; *SUC*2	Lab collection
Q3352	*E. coli* W3110/pACYCDuet1	This study
Q3092	*E. coli* W3110/pACYCDuet-*adhE*_mut_	This study
Q3094	*E. coli* W3110/pACYCDuet-*adhE*_mut_/pBAD-Ae-pha	This study
Q3095	*E. coli* W3110/pACYCDuet1/pBAD-Ae-pha	This study
Q3115	*E. coli* BL21(DE3)/pACYCDuet-*adhE*_mut_/pBAD-Ae-pha	This study
Q3133	*E. coli* W3110/pACYCDuet-*adhE*_mut_-*dnaKJ*/pBAD-Ae-pha	This study
Q3135	*E. coli* W3110/pACYCDuet-*adhE*_mut_-POS5/pBAD-Ae-pha	This study
Q3140	*E. coli* W3110/pACYCDuet-*acs*/pBAD-Ae-pha	This study
Q3397	*E. coli* MG1655 (DE3)/pACYCDuet1/pET-MCRCN	This study
Q3398	*E. coli* MG1655 (DE3)/pACYCDuet1-*adhE*_mut/_pET-MCRCN	This study
Q3399	*E. coli* MG1655 (DE3)/pACYCDuet1-*acs*/pET-MCRCN	This study
Q3432	*E. coli* W3110/pACYCDuet1/pET28a-*phlD*	This study
Q3433	*E. coli* W3110/pACYCDuet1-*adhE*_mut_/pET28a-*phlD*	This study
Q3434	*E. coli* W3110/pACYCDuet1-*acs*/pET28a-*phlD*	This study

**Primers**	**Sequence(5′–3′)**	**Restriction site**

*adhE*-F	CGCGGATCCGATGGCTGTTACTAATGTCGC	*Bam*HI
*adhE*-R	CCGGAGCTCTTAAGCGGATTTTTTCGCTT	*Sac*I
*adhE*(A267T)-F	CGCTGTACGTGAACGTTTTACCACCCACGGCGGCTATCTGTT	–
*adhE*(A267T)-R	AACAGATAGCCGCCGTGGGTGGTAAAACGTTCACGTACAGCG	–
*adhE*(E568K)-F	ATCCGGAAACTCACTTCGAAAAACTGGCGCTGCGCTTTATGGA	–
*adhE*(E568K)-R	TCCATAAAGCGCAGCGCCAGTTTTTCGAAGTGAGTTTCCGGAT	–
*acs*-F	CGCGGATCCGATGAGCCAAATTCACAAACAC	*Bam*HI
*acs*-R	CCGGAGCTCTTACGATGGCATCGCGATAGCC	*Sac*I
*dnaKJ*-F	ATAAGAATGCGGCCGCTTACAGACTCACAACCACAT	*Not*I
*dnaKJ*-R	GGAATTCCATATGTTAGCGGGTCAGGTCGTCAA	*Nde*I
*POS5*-F	ATAAGAATGCGGCCGCTTAATAAGGAGATATACCATGAGTACGTTGGATTCACATTCC	*Not*I
*POS5*-R	GGAATTCCATATGTTAATCATTATCAGTCTGTCTCTTGG	*Nde*I

### Protein Expression and Gel Electrophoresis Analysis

For the expression of recombinant proteins, single colonies of *E. coli* W3110 harboring recombinant plasmids were used to inoculate Luria–Bertani (LB) medium containing appropriate antibiotics and grown at 37°C overnight. The culture was diluted 1:100 into fresh LB medium and incubated under the same conditions. When the OD_600_ reached about 0.6, the protein expression was induced by 100 μM IPTG and growth was continued for 12 h at 30°C. Cells were collected by centrifugation and suspended in 100 μL 1 × SDS sample buffer, heated at 100°C for 10 min and then analyzed by SDS-PAGE.

### Shaking Flask Cultivation

Shaking flask experiments were carried out in triplicate in 250 mL flasks containing 50 mL medium with appropriate antibiotics. For the production of PG and PHB, the medium contains 9.8 g/L K_2_HPO_4_⋅3H_2_O, 2.1 g/L C_6_H_8_O_7_⋅H_2_O, 0.3 g/L ammonium ferric citrate, 3 g/L (NH_4_)_2_SO_4_. For the production of 3HP, the medium contains 14 g/L K_2_HPO_4_⋅3H_2_O, 5.2 g/L KH_2_PO4, 1 g/L NaCl, 1 g/L NH_4_Cl, 0.25 g/L MgSO_4_⋅7H_2_O, 0.2 g/L yeast extract. *E. coli* strains were grown overnight at 37°C with shaking in LB broth, and then 1:50 diluted into 50 mL minimal medium supplemented with corresponding carbon sources. When the OD_600_ reached about 0.6, 100 μM IPTG and 1 g/L L-arabinose was added and the strains are further incubated 48 h at 30°C. Samples were taken out to determine the OD_600_, the concentrations of PHB, 3HP, PG, and residual carbon sources.

### Fed-Batch Fermentation of PHB

Fed-batch cultures were performed in a 3-L fermenter (Applikon Biotechnology, Netherlands) containing 2 L of minimal medium as described above. The strain Q3135 was grown in 150 mL LB broth overnight, and transferred into the fermenter. The temperature was 30°C, the pH was 7.0 and the dissolved oxygen was maintained at 5% saturation. The feeding medium was 50% (w/v) ethanol. The AdhE_mut_ expression was induced at OD_600_ ∼ 3 by addition of 0.1 mM IPTG, and PHB production was started at OD_600_ ∼ 10 by adding 2 g/L arabinose.

### CDW and PHB Content Analysis

*E. coli* cells were harvested with centrifugation at 5,000 × *g* for 15 min, and washed with distilled water twice. To determine CDW, cell pellets were lyophilized, and the CDW was gravimetrically determined. PHB was extracted from lyophilized cells with hot chloroform in a Soxhlet apparatus, and precipitated by icecold ethanol as described (Brandl et al., 1988).

### Quantification of 3HP, PG, Ethanol, Acetate, and Glucose

Concentrations of 3HP, ethanol, acetate and glucose in medium were determined using an Agilent 1200 Infinity HPLC system equipped with an Aminex HPX-87H (Bio-Rad, Hercules, CA, United States) column (300 × 7.8 mm). The concentration of PG was determined using the colorimetric reaction between cinnamaldehyde and PG as previously described (Xu et al., 2017).

### Metabolite Measurements

Metabolites were extracted from seven individual bacteria samples. Bacteria samples were taken 200 μL, then added 800 μL pre-cooled methanol/acetonitrile (1:1, v/v), and mixed in vortex. After sonication for 20 min on ice, the mix was stored at −20°C for 1 h to precipitate proteins. The mix was centrifuged for 15 min (13,000 rpm, 4°C) and supernatant was dried by a vacuum drying system. A targeted metabolic analysis was performed using an LC-MS/MS system. The dried metabolites were dissolved in 100 μL of acetonitrile/H_2_O (1:1, v/v) and centrifuged (14,000 × *g*) for 20 min. Electrospray ionization was conducted with an Agilent 1290 Infinity chromatography system and AB Sciex QTRAP 5500 mass spectrometer. NH_4_COOH (10 mM) and acetonitrile were used as mobile phases A and B, respectively. The sample was placed in a 4°C automatic sampler with a column temperature of 45°C, a flow rate of 300 μL/min and an injection volume of 2 μL. A binary solvent gradient was used as follows: A, NH_4_COOH; B, 0–18 min at 90–40% acetonitrile; 18–18.1 min at 40–90% acetonitrile; and 18.1–23 min at 90% acetonitrile. The LC-MS/MS was operated in the negative mode under the following conditions: source temperature, 450°C; ion source gas 1, 45; ion source gas 2, 45; curtain gas, 30; and ion spray voltage floating, −4,500 V.

## Results and Discussion

### Tolerance of *E. coli* to Ethanol and Acetate

**FIGURE 2:**
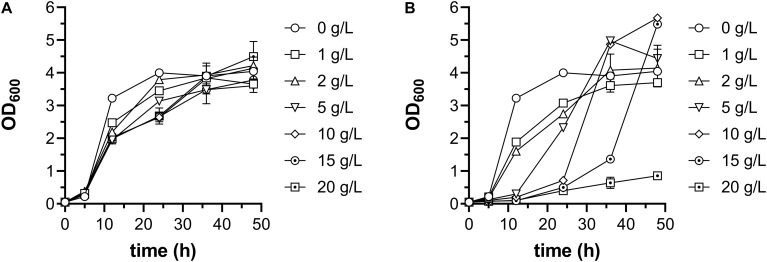
Growth of *E. coli* W3110 strain in minimal medium supplemented with 10 g/L glucose and different concentration of ethanol **(A)** and acetate **(B)**. When acetate was added, pH was adjusted to 7.0 with sodium hydroxide. This experiment was carried out in duplicate, and data represent mean ± SEM.

Ethanol usually shows antimicrobial activity at high concentration, and acetate represses the growth rate and maximum cell density of *E. coli* (Liu et al., 2015). To figure out appropriate concentrations of ethanol and acetate as carbon source, they were added into minimal medium containing 10 g/L glucose at a series of concentrations from 1 to 20 g/L, respectively. As shown in Figure 2, addition of ethanol slightly slowed the growth, but did not affect maximum cell density of *E. coli* W3110 wild-type strain, as well as acetate at concentrations of 1 and 2 g/L. However, acetate at concentrations of 5–15 g/L severely retarded *E. coli* growth, and 20 g/L acetate almost inhibited bacterial growth. As *E. coli* can catabolize acetate naturally, the maximum cell density with addition of 5–15 g/L acetate was higher than that at control conditions. Therefore, the concentration of ethanol was fixed at 10 g/L, and 2 g/L acetate was supplied at the beginning of cultivation with follow-up addition when exhausted.

### Construction of *E. coli* Strain Growing on Ethanol

**FIGURE 3:**
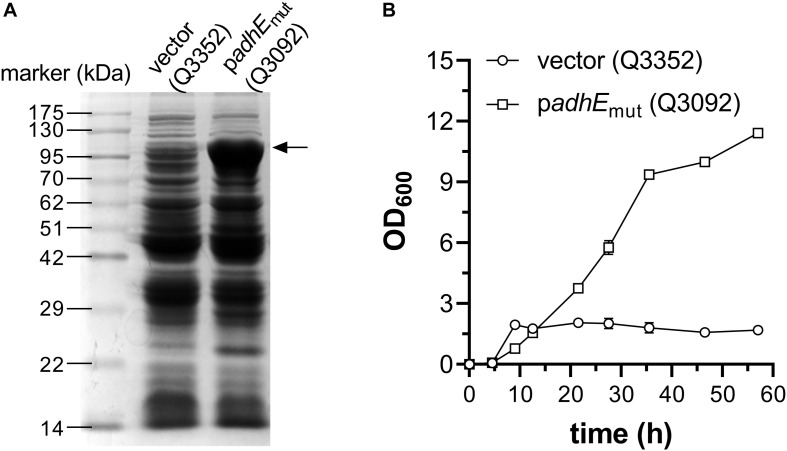
Construction of *E. coli* strain growing on ethanol as sole carbon source. **(A)** Expression of acetyl-CoA reductase AdhE mutant in *E. coli* cells. Crude cell extracts of *E. coli* W3110 strains carrying empty vector (Q3352) or recombinant plasmid for overexpression of AdhE A267T/E568K mutant (Q3092) were used. The position of overexpressed AdhE mutant (98 kDa) is indicated by arrow. **(B)** Growth profile of these strains in minimal medium supplemented with 1 g/L glucose and 10 g/L ethanol. This experiment was carried out in triplicate, and data represent mean ± SEM.

As mentioned above, two systems, *E. coli* AdhE mutant and *P. aeruginosa* ExaABC, can support bacterial growth on ethanol as sole carbon source (Figure 1B). As the route employing AdhE mutant has some advantages like shorter pathway, more NADH production and no energy requirement, it was chosen to construct engineered *E. coli* strain which can grow using ethanol as sole carbon source. The gene encoding AdhE A267T/E568K mutant was cloned into the vector pACYCDuet-1 under the IPTG-inducible promoter P*_*lac*_*_1__–__6_ (Liu et al., 2004), and transformed into *E. coli* W3110 strain, along with empty vector, to generate strains Q3092 and Q3352, respectively. When grown in LB broth and induced by IPTG, AdhE mutant protein was observed as distinct band with the expected molecular weight on SDS-PAGE (Figure 3A).

Then, these two strains were inoculated into minimal medium supplemented with 10 g/L ethanol, but neither could grow under this condition, implying that overexpression of heterologous gene at beginning of cultivation might impair the cell growth seriously. So, 1 g/L glucose was added into the medium, and transcription of *adhE*_mut_ gene was initiated at an OD_600_ of 0.6. After 57 h cultivation in shaking flask, expression of AdhE mutant significantly increased the cell density, up to 6.7-time higher than the strain carrying empty vector (Figure 3B), suggesting that appropriate expression of AdhE double mutant is efficient to support *E. coli* growth on ethanol.

### Production of PHB From Ethanol

To produce PHB from ethanol, the plasmid pBAD-Ae-pha carrying *phaCAB* operon of *C. necator* (Aldor and Keasling, 2001) was transformed into strains Q3352 and Q3092 to generate Q3095 and Q3094, respectively. Then, these two strains were grown in minimal medium containing 1 g/L glucose and 10 g/L ethanol, and expression of *adhE*_mut_ and *phaCAB* was initiated by addition of IPTG and L-arabinose, respectively. During the cultivation process, the OD_600_ and residual ethanol concentration in medium were measured.

**FIGURE 4:**
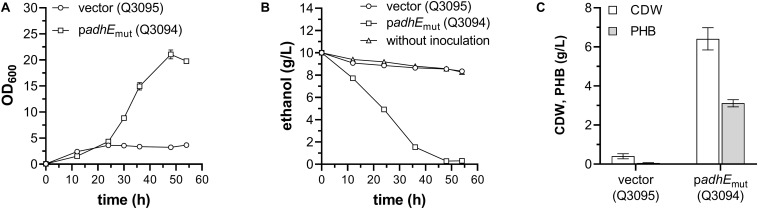
PHB production of engineered *E. coli* W3110 strain with ethanol as sole carbon source. **(A)** The growth profile, **(B)** residual ethanol in medium, and **(C)** cell dry weight (CDW) and PHB production of *E. coli* W3110 strains carrying PHB-producing plasmid pBAD-Ae-pha in addition to the empty vector (Q3095) or AdhE_mut_ plasmid (Q3094). Minimal medium used in these experiments is supplemented with 1 g/L glucose and 10 g/L ethanol. These experiments were carried out in triplicate, and data represent mean ± SEM.

As shown in Figure 4A, the final OD_600_ of Q3094 strain carrying AdhE mutant was much higher than that of the control strain Q3095 carrying empty vector. Growth of Q3094 strain depleted all ethanol in the medium, however, the ethanol concentration in Q3095 culture decreased slightly, similar with that of medium without inoculation but incubated at 37°C (Figure 4B). After 54 h cultivation, the strain Q3094 accumulated 3.12 ± 0.15 g/L PHB representing 48.7% of cell dry weight (CDW), while the control strain Q3095 produced 0.40 ± 0.11 g/L CDW and trace amount of PHB (Figure 4C). All these results demonstrated that *E. coli* with overexpression of AdhE_mut_ acquired the ability to produce value-added bioproducts using ethanol as carbon source.

### Comparison of Glucose, Ethanol and Acetate as Carbon Source for PHB Production

**FIGURE 5:**
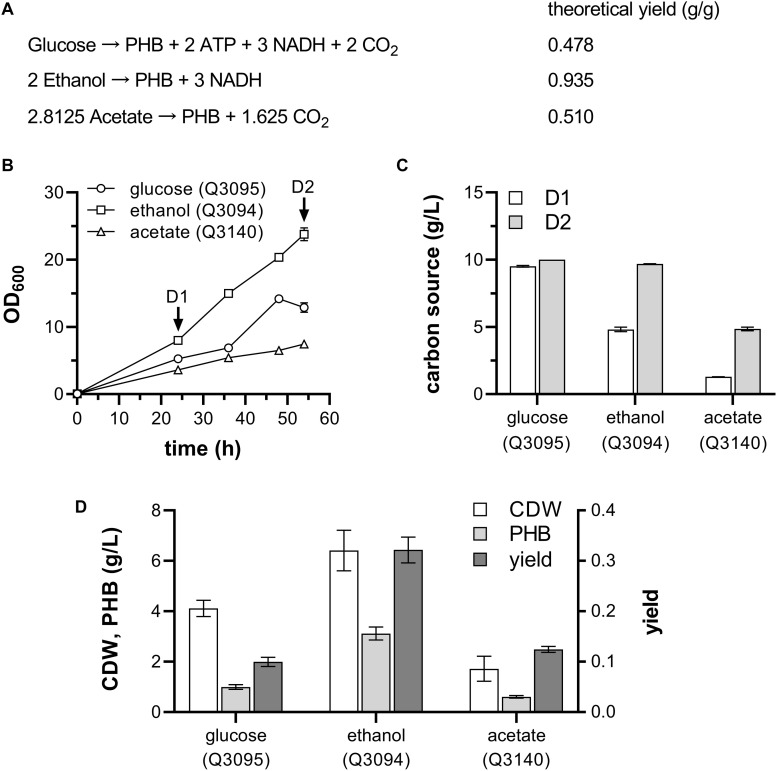
PHB production from glucose, ethanol and acetate by engineered *E. coli.* **(A)** Theoretical yield of PHB from different carbon sources. **(B)** Growth curve. **(C)** Carbon source consumed by various strains at D1 and D2 stage in panel **(B)**. **(D)** Cell dry weight, PHB production and yield by different *E. coli* strains. Three strains carrying PHB-producing plasmid pBAD-Ae-pha in addition to the empty vector (Q3095), AdhE plasmid (Q3094), and ACS plasmid (Q3140) were grown with glucose, ethanol, and acetate as carbon source, respectively. For Q3095 and Q3094, 10 g/L glucose and 10 g/L ethanol are supplemented in addition of 1 g/L glucose, respectively. For Q3140, 1 g/L glucose and 2 g/L acetate were added into the medium at 0 h, and 3 g/L acetate was added at D1 stage. These experiments were carried out in triplicate, and data represent mean ± SEM.

Combining the reactions in Figures 1A,B, the overall theoretical stoichiometry of PHB synthesis from various carbon source is deduced (Figure 5A). It shows that the PHB production from both glucose and ethanol is coupled with generation of energy and reducing power, but extra acetate is oxidized to meet the demand of energy and reducing power in pathway from acetate to PHB. The theoretical yield of PHB from ethanol is 0.935 g/g, much higher than its theoretical yields from the other two feedstocks. So, it is hypothesized that ethanol could be an ideal carbon source for PHB production in engineered bacteria.

To verify this hypothesis, comparison of glucose, ethanol and acetate as carbon source for PHB production was conducted. The gene *acs* was cloned into the vector pACYCDuet-1 under the IPTG-inducible promoter P*_*lac*_*_1__–__6_, and co-transformed with pBAD-Ae-pha into *E. coli* W3110 to generate strain Q3140. Then Q3095, Q3094 and Q3140 were grown on glucose, ethanol and acetate, respectively, and the cell growth, consumption of carbon source, PHB production, and intracellular metabolites were monitored during the cultivation process.

After 54 h cultivation, the strain Q3094 presented the highest cell density, followed by strain Q3095 and Q3140 (Figure 5B). At D1 and D2 stages shown in Figure 5B, the consumption of carbon sources was measured. The strain Q3095 almost exhausted 10 g/L glucose in 24 h, while 4.81 ± 0.17 g/L ethanol and 1.29 ± 0.01 g/L acetate were consumed by strain Q3094 and Q3140, respectively. At the end of cultivation, the strain Q3094 consumed 9.68 ± 0.02 g/L ethanol, and 4.85 ± 0.13 g/L acetate was catabolized by strain Q3140 (Figure 5C). The PHB production of Q3094 is 3.12 ± 0.18 g/L, and the yield of PHB from ethanol is 0.32 ± 0.02 g/g ethanol. Both PHB production and yield were much higher than those from glucose and acetate (Figure 5D). These results suggested that ethanol have some advantages as carbon source for PHB production than glucose and acetate, such as high cell density, high production and yield.

### Differences Between the Metabolism of Glucose, Ethanol, and Acetate

**FIGURE 6:**
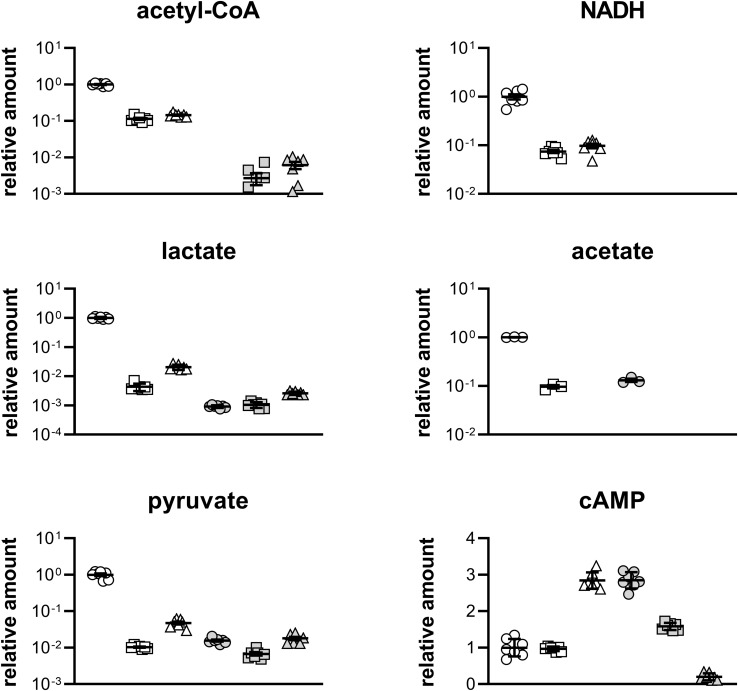
Relative quantification of metabolites in different strains at D1 and D2 stages in Figure 5B. Circle, Q3095 grown on glucose; square, Q3094 grown on ethanol; triangle, Q3140 grown on acetate; open symbol, D1 stage; filled symbol, D2 stage. These experiments were carried out in septuplicate, and data represent mean ± SEM. The acetate production of Q3140 strain cannot be determined as acetate was added as carbon source.

To figure out the differences between the metabolism of glucose, ethanol and acetate, the targeted metabolome analysis was performed. Relative amount of acetyl-CoA, by-products, and main metabolites in central carbon metabolism pathways was determined at D1 and D2 stages (Figure 5B) by LC-MS/MS, and shown in Figure 6.

At D1 stage, Q3095 strain grown on glucose produced the most acetyl-CoA, which was 8.73 times and 6.91 times higher than strains Q3094 and Q3140, respectively, and the similar results were observed in supply of energy and reducing power (Figure 6 and Supplementary Figure S1). It is probably because that glucose is the favorite carbon source of *E. coli* (Gorke and Stulke, 2008), and was rapidly metabolized. This was also confirmed by the highest consumption rate of glucose (Figure 5C). At D2 stage, acetyl-CoA and NADH were undetectable in Q3095 strain as glucose had been depleted.

For metabolites in glycolysis pathway, Q3095 strain also showed the highest abundance at D1 stage. In particular, the pyruvate amount in Q3095 strain was more than 20 times higher than those in the other two strains (Figure 6 and Supplementary Figure S1). It was very reasonable because that glucose was metabolized mainly through glycolysis pathway, and in Q3094 and Q3140 strains these metabolites have to be produced from ethanol and acetate through gluconeogenesis as building blocks for synthesis of components for construction of cell architecture. At D2 stage, there was not significant difference between three strains.

In contrast, the relative amount of TCA cycle intermediates in Q3140 strain was significantly higher than those in the other two strains at both D1 and D2 stages (Supplementary Figure S1). This phenomenon proved our hypothesis that extra acetate was oxidized through TCA cycle to produce ATP and NADH.

Remarkably, two by-products, lactate and acetate, were produced in Q3095 strain in large quantities at D1 stage (Figure 6), and the accumulation of acidic products could inhibit further growth of Q3095 strain. The lactate production of Q3095 strain was 229.22 times and 48.59 times as much as those of Q3094 strain and Q3140 strain, respectively. The accumulation of lactate was consistent with the high contents of pyruvate and NADH in Q3095 strain. As is known, glucose is catabolized through glycolysis pathway to pyruvate, which is converted into acetyl-CoA under catalysis of pyruvate dehydrogenase, with reduction of NAD^+^ to NADH in both glycolysis pathway and pyruvate dehydrogenation. When NADH could not be oxidized through respiratory chain sufficiently, the build-up of NADH rapidly inactivated the pyruvate dehydrogenase (Hansen and Henning, 1966), leading to accumulation of pyruvate in cells, and the recycling of NAD^+^ must be achieved through the reduction of some metabolites. Given the circumstances, a large quantity of lactate was produced by lactate dehydrogenase LdhA via reduction of pyruvate with consumption of NADH molecule (Bunch et al., 1997).

Furthermore, Q3095 strain accumulated acetate 10.33-fold higher than Q3094 strain at D1 stage. In addition to be oxidized through TCA cycle, acetyl-CoA could be converted into acetate by PTA and AckA, in coupling with generation of one ATP molecule (Fox and Roseman, 1986). It was demonstrated that the majority of the acetyl-CoA is converted into acetate through the PTA-AckA pathway, and only a minority is metabolized via TCA cycle to generate NADH and CO_2_ when *E. coli* grows on glucose aerobically (Clark, 1989). Therefore, acetate accumulated in medium in large amount. Acetate overflow is caused by an imbalance between the pathways of glycolysis and TCA cycle in rapidly growing cells, and severely decreases the yield of target chemicals from glucose (Farmer and Liao, 1997; Wong et al., 2008). When the glucose supply is depleted, lactate and acetate can be taken back into the cells and respired. So, the amount of lactate and acetate dramatically decreased at D2 stage. Based on the above, the growth of *E. coli* on glucose was classically a diauxie, consuming glucose in the first half and consuming lactate and acetate in the second half. It was reported that *E. coli* cells grown on glucose produce acetate and consume it after glucose exhaustion, but do not grow on acetate due to the decoupling of acetate anabolism and acetate catabolism, and the growth restores only after prolonged exposure to acetate (Enjalbert et al., 2015). Here, it is believed that this glucose-lactate/acetate transition would delay the growth of *E. coli*, and further affect the production of target product.

Another thing that deserves special attention was that the levels of cyclic AMP (cAMP) in Q3095 and Q3094 strains were essentially the same, much lower than that in Q3140 strain grown on acetate at D1 stage (Figure 6). The secondary messenger cAMP is synthesized from ATP, catalyzed by the adenylyl cyclase Cya whose activity is controlled by glucose availability (Notley-McRobb et al., 1997). When the preferred carbon source glucose is absent, the intracellular cAMP concentration increases ∼10-fold, converting the cAMP receptor protein CRP into an active form to activate a number of genes for utilization of carbon sources other than glucose (Fic et al., 2009). The low cAMP level in Q3094 strain grown on ethanol indicated that ethanol could be one of preferred carbon sources of *E. coli.*

### Optimization of Recombinant *E. coli* Strain Producing PHB From Ethanol

**FIGURE 7:**
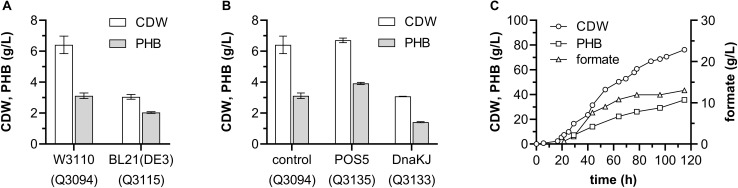
Optimization of the *E. coli* recombinant strain producing PHB from ethanol. **(A)** Effect of host strains on PHB production from ethanol. **(B)** Effect of NADH kinase POS5 and chaperone DnaKJ on PHB production from ethanol. **(C)** Time profiles for CDW and PHN production during an aerobic fed-batch fermentation of the Q3135 strain. These experiments shown in panels **(A,B)** were carried out in duplicate, and data represent mean ± SEM. **(C)** shows a representative result from two independent experiments.

As the engineered *E. coli* strain producing PHB from ethanol presented the highest production and yield, further development of this strain was carried out. To evaluate the potential influences of different host strains on PHB production, plasmids pBAD-Ae-pha and pACYCDuet-*adhE*_mut_ were transformed into *E. coli* BL21(DE3) to generate the strain Q3115, which was compared with the W3110-based strain Q3094. Grown in minimal medium containing 1 g/L glucose and 10 g/L ethanol, the strain Q3115 accumulated 2.04 ± 0.05 g/L PHB, which was much lower than that of Q3094 strain (Figure 7A).

The chaperone DnaK was proved to protect the mutant AdhE against metal-catalyzed oxidation and to improve the aerobic growth on ethanol of *E. coli* (Echave et al., 2002). Furthermore, NADH kinase (POS5) from *S. cerevisiae* generates NADPH from NADH phosphorylation, helping the cofactor regeneration during PHB synthesis (Hong et al., 2016). So, the genes *dnaKJ* and *POS5* were cloned and tested, respectively. As shown in Figure 7B, POS5 overexpression increased the PHB production from ethanol to 3.92 ± 0.06 g/L, representing 58.5% of CDW, however, the introduction of *dnaKJ* impaired the PHB synthesis severely.

Then, the strain Q3135 was subjected to fed-batch fermentation to test the feasibility of PHB production from ethanol in large scale. The fermentations were carried out under aerobic conditions using minimal medium containing 1 g/L glucose and 10 g/L ethanol, and ethanol was fed when the carbon source was depleted. After 115-h fermentation, the strain Q3135 produced 35.67 g/L PHB representing 46.8% of CDW, and 130.5 g/L ethanol was consumed, achieving a yield of 0.273 g/g ethanol (Figure 7C). It was worth noting that the byproduct formate was accumulated to a concentration of 13.0 g/L, and the genes involved in formate biosynthesis could be the metabolic engineering target to improve the PHB yield.

### Production of Other Acetyl-CoA Derivatives From Ethanol

**FIGURE 8:**
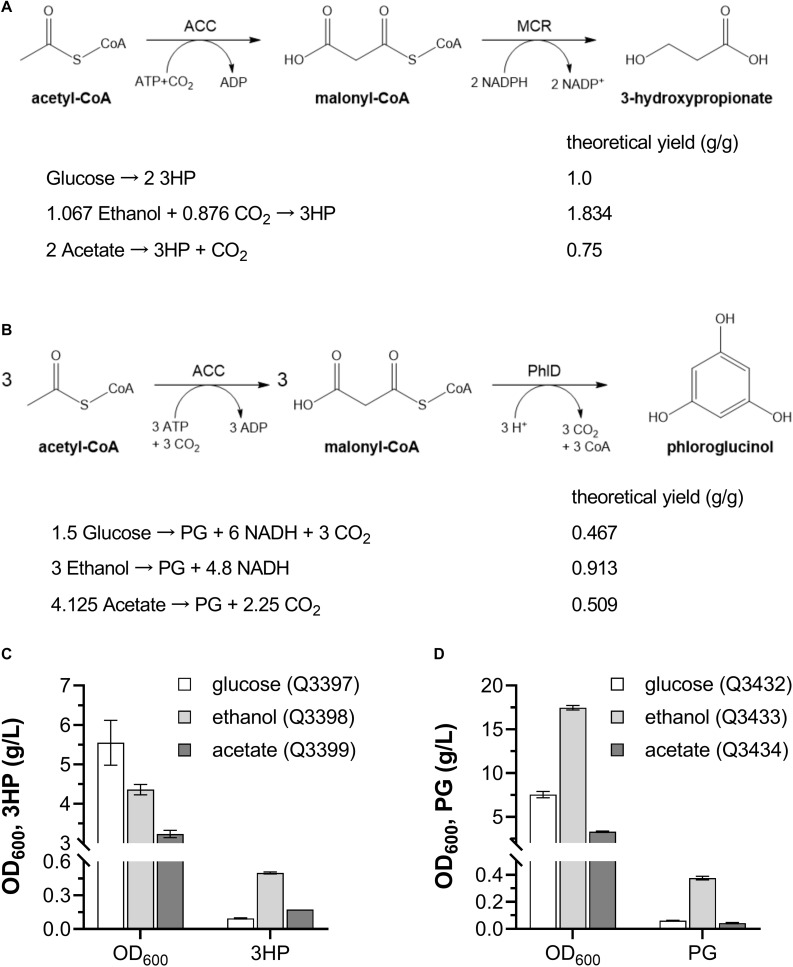
Production of 3HP and PG from various carbon sources by engineered *E. coli* strains. **(A)** 3HP biosynthetic pathway and theoretical yield from different carbon sources. **(B)** PG biosynthetic pathway and theoretical yield from different carbon sources. **(C)** Cell density and 3HP production when grown with glucose, ethanol and acetate as sole carbon source. **(D)** Cell density and PG production when grown with glucose, ethanol and acetate as sole carbon source. ACC, acetyl-CoA reductase; MCR, malonyl-CoA reductase; PhlD, polyketide synthase. These experiments were carried out in triplicate, and data represent mean ± SEM.

To test whether ethanol is suitable for production of other acetyl-CoA derived chemicals, the biosynthetic pathways for PG and 3HP from glucose, ethanol and acetate were constructed in *E. coli*, respectively, and comparison of those three carbon sources was carried out. 3HP and PG are both derived from the intermediate malonyl-CoA, which is produced from acetyl-CoA under catalysis of acetyl-CoA carboxylase ACC. Then malonyl-CoA is reduced to 3HP by malonyl-CoA reductase MCR (Figure 8A), or catalyzed by polyketide synthase PhlD to form PG (Figure 8B). The theoretical yields of 3HP and PG from various carbon sources were calculated, and those from ethanol were the highest (Figures 8A,B).

Then, the 3HP- and PG-producing *E. coli* strains from glucose, ethanol and acetate were constructed, respectively, and were inoculated into minimal medium with corresponding carbon sources. After cultivation in shaking flasks, Q3398 strain grown on ethanol produced 0.50 ± 0.01 g/L 3HP (Figure 8C), and Q3433 strain grown on ethanol produced 0.38 ± 0.02 g/L PG (Figure 8D), much higher than strains grown on the other two carbon sources. These results demonstrate that ethanol can be used as carbon source for production of acetyl-CoA derived chemicals besides PHB.

### Ethanol Is a Suitable Carbon Source for Production of Acetyl-CoA Derivatives

Acetyl-CoA is a fundamental metabolite in bacterial central metabolic pathways, and also a precursor for biosynthesis of large number of materials and chemicals (Martin et al., 2003; Meng et al., 2012; Liu et al., 2016). A series of acetyl-CoA derived chemical and material have been successfully produced by engineered *E. coli* strains, such as PG (Cao et al., 2011), 3HP (Liu et al., 2016, 2019), fatty acids (Cao et al., 2010), polyketides (Yuzawa et al., 2012), terpenoids (Martin et al., 2003), and PHAs (Meng et al., 2012).

Conversion of glucose into acetyl-CoA presents low atomic economy due to the release of CO_2_, leading to decrease of theoretical production yield, titer and productivity of target product (Chae et al., 2017). To improve the atomic economy of biosynthesis process, fatty acids were tested as an alternative carbon source (Liu et al., 2019). Catabolized through β-oxidation pathways, fatty acids are transformed into acetyl-CoA with 100% carbon recovery in addition to production of reducing power. Although the production and yield of target chemical were increased significantly (Liu et al., 2019), the water-insoluble nature of fatty acids makes it difficult to handle and monitor the fermentation process. Several synthetic pathways for acetyl-CoA from one-carbon substrates have been constructed in *E. coli* (Lu et al., 2019; Wang et al., 2019), but their efficiency remains far below what is required for effective production of chemicals and materials.

In this study, *E. coli* strain growing on ethanol was constructed, and the bioconversion of ethanol into a series of bioproducts including PHB, 3HP and PG was achieved in recombinant *E. coli* strains. Furthermore, metabolome analysis was carried out to discover the differences between metabolism of glucose, ethanol and acetate in engineered *E. coli* strains. All these results suggested that ethanol may be a suitable carbon source for production of acetyl-CoA derived bioproducts.

Firstly, ethanol is an ordinary and inexpensive commodity chemical. Besides fermentation of lignocellulosic biomass (Sun and Cheng, 2002), ethanol can also be produced from coal by chemical methods. The capacity of ethanol production from coal has exceeded 2 million tons per year in China. Secondly, the conversion of ethanol into acetyl-CoA presents high atomic economy, in addition to the generation of NADH. However, CO_2_ is released in glucose catabolism and ATP is consumed to synthesis acetyl-CoA from acetate (Figure 1). Therefore, the theoretical yield of target chemical from ethanol is much higher than those from glucose and acetate (Figures 5A, 7A,B). Furthermore, ethanol has a higher energy density. If oxidized completely to CO_2_ and H_2_O in bacteria, 0.326 mol of ATP is generated from 1 g ethanol, while 0.178 and 0.133 mol of ATP can be produced from 1 g glucose and acetate, respectively. Moreover, *E. coli* is easy to reach a balance between growth and production when grown on ethanol. As glucose is the favorite carbon source of *E. coli*, it was rapidly metabolized in the first half of cultivation (Figure 5C) to produce a large amount of lactate and acetate (Figure 6), leading to a bi-phasic growth which may delay the further bacterial growth and lower the production of target chemical. The assimilation of ethanol was neither as fast as glucose to accumulate by-products, nor as slow as acetate to retard the bacterial growth, helping bacteria reach a balance between growth and production. Additionally, the nature of ethanol, such as low toxicity and high water-solubility, makes it friendly to the bacterial cultivation process.

Although our study showed the feasibility of ethanol to support *E. coli* growth and bioproduction, there are still some problems remaining. The ethanol utilizing gene *adhE*_mut_ was carried by a plasmid vector, leading to addition of antibiotic into medium and increasing of production cost. Besides that, the strain performance may be affected by the strain instability due to plasmid loss. This problem can be dissolved by the integration of *adhE*_mut_ gene into bacterial chromosome. Furthermore, the PHB yield was 0.32 ± 0.02 g/g ethanol in our engineered strain, representing only 34% of the theoretical limit (Figure 4), and the productions of 3HP and PG from ethanol were both much lower than previous reports from other carbon sources probably due to the lack of overexpression of acetyl-CoA carboxylase (Cao et al., 2011; Liu et al., 2019). It is necessary to carry out further development to achieve a higher production and yield from ethanol.

### Conclusion

In summary, the bifunctional dehydrogenase AdhE A267T/E568K mutant, which converts ethanol into acetyl-CoA, was introduced into *E. coli* strain, conferring *E. coli* the growth capability on ethanol. Several products derived from acetyl-CoA, such as PHB, 3HP and PG, were produced from ethanol by recombinant *E. coli* strains. Compared with glucose and acetate, the strains grown on ethanol presented the highest production and yield, and metabolome analysis revealed the reasons of high yield from ethanol. All these results demonstrate that ethanol is a putative carbon source for production of acetyl-CoA derived bioproducts.

## Data Availability Statement

The original contributions presented in the study are included in the article/Supplementary Material, further inquiries can be directed to the corresponding author.

## Author Contributions

GZ and MX designed the study. SS, YD, and ML performed the strain construction, fermentation, and metabolome analysis. SS, ML, MX, and GZ analyzed the data. SS and GZ wrote the manuscript. All authors read and approved the final manuscript.

## Conflict of Interest

The authors declare that the research was conducted in the absence of any commercial or financial relationships that could be construed as a potential conflict of interest.

## Abbreviations

3HP: 3-hydroxypropionate
ACS: AMP-forming acetyl-CoA synthetase
cAMP: cyclic AMP
CDW: cell dry weight
PG: phloroglucinol
PHB: poly(3-hydroxybutyrate)
TCA cycle: tricarboxylic acid cycle.

## References

[B1] Aldor I. Keasling J. D. (2001). Metabolic engineering of poly(3-hydroxybutyrate-co-3-hydroxyvalerate) composition in recombinant *Salmonella enterica* serovar typhimurium. *Biotechnol. Bioeng.* 76 108–114. 10.1002/bit.1150 11505380

[B2] Bates D. L. Danson M. J. Hale G. Hooper E. A. Perham R. N. (1977). Self-assembly and catalytic activity of the pyruvate dehydrogenase multienzyme complex of *Escherichia coli*. *Nature* 268 313–316. 10.1038/268313a0 329143

[B3] Brandl H. Gross R. A. Lenz R. W. Fuller R. C. (1988). *Pseudomonas oleovorans* as a source of poly(β-hydroxyalkanoates) for potential applications as biodegradable polyesters. *Appl. Environ. Microbiol.* 54 1977–1982. 10.1128/aem.54.8.1977-1982.1988 16347708 PMC202789

[B4] Bunch P. K. Mat-Jan F. Lee N. Clark D. P. (1997). The ldhA gene encoding the fermentative lactate dehydrogenase of *Escherichia coli*. *Microbiology* 143 (Pt 1), 187–195. 10.1099/00221287-143-1-187 9025293

[B5] Cao Y. J. Jiang X. L. Zhang R. B. Xian M. (2011). Improved phloroglucinol production by metabolically engineered *Escherichia coli*. *Appl. Microbiol. Biotechnol.* 91 1545–1552. 10.1007/s00253-011-3304-5 21643705

[B6] Cao Y. J. Yang J. M. Xu X. Liu W. Xian M. (2010). Increasing unsaturated fatty acid contents in *Escherichia coli* by coexpression of three different genes. *Appl. Microbiol. Biotechnol.* 87 271–280. 10.1007/s00253-009-2377-x 20135119

[B7] Chae T. U. Choi S. Y. Kim J. W. Ko Y. S. Lee S. Y. (2017). Recent advances in systems metabolic engineering tools and strategies. *Curr. Opin. Biotechnol.* 47 67–82. 10.1016/j.copbio.2017.06.007 28675826

[B8] Chen G. Q. (2009). A microbial polyhydroxyalkanoates (PHA) based bio- and materials industry. *Chem. Soc. Rev.* 38 2434–2446. 10.1039/b812677c 19623359

[B9] Chen Y. M. Lin E. C. (1991). Regulation of the adhE gene, which encodes ethanol dehydrogenase in *Escherichia coli*. *J. Bacteriol.* 173 8009–8013. 10.1128/jb.173.24.8009-8013.1991 1744060 PMC212600

[B10] Clark D. P. (1989). The fermentation pathways of *Escherichia coli*. *FEMS Microbiol. Rev.* 63 223–234. 10.1016/0168-6445(89)90033-8 2698228

[B11] Diehl A. von Wintzingerode F. Gorisch H. (1998). Quinoprotein ethanol dehydrogenase of *Pseudomonas aeruginosa* is a homodimer - Sequence of the gene and deduced structural properties of the enzyme. *Eur. J. Biochem.* 257 409–419. 10.1046/j.1432-1327.1998.2570409.x 9826187

[B12] Echave P. Esparza-Ceron M. A. Cabiscol E. Tamarit J. Ros J. Membrillo-Hernandez J. (2002). DnaK dependence of mutant ethanol oxidoreductases evolved for aerobic function and protective role of the chaperone against protein oxidative damage in *Escherichia coli*. *Proc. Natl. Acad. Sci. U.S.A.* 99 4626–4631. 10.1073/pnas.072504199 11917132 PMC123698

[B13] Enjalbert B. Cocaign-Bousquet M. Portais J. C. Letisse F. (2015). Acetate exposure determines the diauxic behavior of *Escherichia coli* during the glucose-acetate transition. *J. Bacteriol.* 197 3173–3181. 10.1128/JB.00128-15 26216845 PMC4560281

[B14] Farmer W. R. Liao J. C. (1997). Reduction of aerobic acetate production by *Escherichia coli*. *Appl. Environ. Microbiol.* 63 3205–3210. 10.1128/aem.63.8.3205-3210.1997 9251207 PMC168618

[B15] Fic E. Bonarek P. Gorecki A. Kedracka-Krok S. Mikolajczak J. Polit A. (2009). cAMP receptor protein from *Escherichia coli* as a model of signal transduction in proteins–a review. *J. Mol. Microbiol. Biotechnol.* 17 1–11. 10.1159/000178014 19033675

[B16] Fox D. K. Roseman S. (1986). Isolation and characterization of homogeneous acetate kinase from *Salmonella typhimurium* and *Escherichia coli*. *J. Biol. Chem.* 261 13487–13497. 3020034

[B17] Gorke B. Stulke J. (2008). Carbon catabolite repression in bacteria: many ways to make the most out of nutrients. *Nat. Rev. Microbiol.* 6 613–624. 10.1038/nrmicro1932 18628769

[B18] Hansen H. G. Henning U. (1966). Regulation of pyruvate dehydrogenase activity in *Escherichia coli* K12. *Biochim. Biophys. Acta* 122 355–358. 10.1016/0926-6593(66)90076-2 4291045

[B19] Hong P. H. Zhang J. Liu X. J. Tan T. W. Li Z. J. (2016). Effect of NADH kinase on poly-3-hydroxybutyrate production by recombinant *Escherichia coli*. *J. Biosci. Bioeng.* 122 685–688. 10.1016/j.jbiosc.2016.06.005 27353858

[B20] Kessler D. Leibrecht I. Knappe J. (1991). Pyruvate-formate-lyase-deactivase and acetyl-CoA reductase activities of *Escherichia coli* reside on a polymeric protein particle encoded by adhE. *FEBS Lett.* 281 59–63. 10.1016/0014-5793(91)80358-a 2015910

[B21] Liu B. Xiang S. Zhao G. Wang B. Ma Y. Liu W. (2019). Efficient production of 3-hydroxypropionate from fatty acids feedstock in *Escherichia coli*. *Metab. Eng.* 51 121–130. 10.1016/j.ymben.2018.10.003 30343047

[B22] Liu C. Ding Y. Zhang R. Liu H. Xian M. Zhao G. (2016). Functional balance between enzymes in malonyl-CoA pathway for 3-hydroxypropionate biosynthesis. *Metab. Eng.* 34 104–111. 10.1016/j.ymben.2016.01.001 26791242

[B23] Liu M. Feng X. Ding Y. Zhao G. Liu H. Xian M. (2015). Metabolic engineering of *Escherichia coli* to improve recombinant protein production. *Appl. Microbiol. Biotechnol.* 99 10367–10377. 10.1007/s00253-015-6955-9 26399416

[B24] Liu M. Tolstorukov M. Zhurkin V. Garges S. Adhya S. (2004). A mutant spacer sequence between -35 and -10 elements makes the Plac promoter hyperactive and cAMP receptor protein-independent. *Proc. Natl. Acad. Sci. U.S.A.* 101, 6911–6916 10.1073/pnas.0401929101 15118087 PMC406441

[B25] Liu Y. Zhu W. Liu H. Ni Y. Liu Z. Meng S. (2013). “Method for producing ethanol and coproducing methanol,” in *United States patent application US15/103,076* (Washington, DC: The United States Patent and Trademark Office).

[B26] Lu X. Liu Y. Yang Y. Wang S. Wang Q. Wang X. (2019). Constructing a synthetic pathway for acetyl-coenzyme A from one-carbon through enzyme design. *Nat. Commun.* 10:1378. 10.1038/s41467-019-09095-z 30914637 PMC6435721

[B27] Martin V. J. Pitera D. J. Withers S. T. Newman J. D. Keasling J. D. (2003). Engineering a mevalonate pathway in *Escherichia coli* for production of terpenoids. *Nat. Biotechnol.* 21 796–802. 10.1038/nbt833 12778056

[B28] McGinnis J. L. (1983). “Direct hydrogenation of carboxylic acids to alcohol and esters,” in *United States patent application US06/476,310* (Washington, DC: The United States Patent and Trademark Office).

[B29] Membrillo-Hernandez J. Echave P. Cabiscol E. Tamarit J. Ros J. Lin E. C. (2000). Evolution of the adhE gene product of *Escherichia coli* from a functional reductase to a dehydrogenase. Genetic and biochemical studies of the mutant proteins. *J. Biol. Chem.* 275 33869–33875. 10.1074/jbc.M005464200 10922373

[B30] Meng D. C. Shi Z. Y. Wu L. P. Zhou Q. Wu Q. Chen J. C. (2012). Production and characterization of poly(3-hydroxypropionate-co-4-hydroxybutyrate) with fully controllable structures by recombinant *Escherichia coli* containing an engineered pathway. *Metab. Eng.* 14 317–324. 10.1016/j.ymben.2012.04.003 22561235

[B31] Notley-McRobb L. Death A. Ferenci T. (1997). The relationship between external glucose concentration and cAMP levels inside *Escherichia coli*: implications for models of phosphotransferase-mediated regulation of adenylate cyclase. *Microbiology* 143 (Pt 6), 1909–1918. 10.1099/00221287-143-6-1909 9202467

[B32] Pan X. Fan Z. Chen W. Ding Y. Luo H. Bao X. (2007). Enhanced ethanol production inside carbon-nanotube reactors containing catalytic particles. *Nat. Mater.* 6 507–511. 10.1038/nmat1916 17515914

[B33] Schobert M. Gorisch H. (1999). Cytochrome c550 is an essential component of the quinoprotein ethanol oxidation system in *Pseudomonas aeruginosa*: cloning and sequencing of the genes encoding cytochrome c550 and an adjacent acetaldehyde dehydrogenase. *Microbiology* 145 471–481. 10.1099/13500872-145-2-471 10075429

[B34] Steinbuchel A. Schlegel H. G. (1991). Physiology and molecular genetics of poly(β-hydroxy-alkanoic acid) synthesis in *Alcaligenes eutrophus*. *Mol. Microbiol.* 5 535–542. 10.1111/j.1365-2958.1991.tb00725.x 2046547

[B35] Sun S. Ding Y. Liu M. Xian M. Zhao G. (2020). Comparison of glucose, acetate and ethanol as carbon source for production of acetyl-CoA derived chemicals in engineered *Escherichia coli*. *Res. Square* [Preprint] 10.21203/rs.3.rs-17185/v1

[B36] Sun Y. Cheng J. (2002). Hydrolysis of lignocellulosic materials for ethanol production: a review. *Bioresour. Technol.* 83 1–11. 10.1016/s0960-8524(01)00212-7 12058826

[B37] Tamarit J. Cabiscol E. Ros J. (1998). Identification of the major oxidatively damaged proteins in *Escherichia coli* cells exposed to oxidative stress. *J. Biol. Chem.* 273 3027–3032. 10.1074/jbc.273.5.3027 9446617

[B38] Wang X. Wang X. Lu X. Ma C. Chen K. Ouyang P. (2019). Methanol fermentation increases the production of NAD(P)H-dependent chemicals in synthetic methylotrophic *Escherichia coli*. *Biotechnol. Biofuels* 12:17. 10.1186/s13068-019-1356-4 30679956 PMC6340170

[B39] Wolfe A. J. (2005). The acetate switch. *Microbiol. Mol. Biol. Rev.* 69 12–50. 10.1128/MMBR.69.1.12-50.2005 15755952 PMC1082793

[B40] Wong M. S. Wu S. Causey T. B. Bennett G. N. San K. Y. (2008). Reduction of acetate accumulation in *Escherichia coli* cultures for increased recombinant protein production. *Metab. Eng.* 10 97–108. 10.1016/j.ymben.2007.10.003 18164227

[B41] Wu H. Chen J. Chen G. Q. (2016a). Engineering the growth pattern and cell morphology for enhanced PHB production by *Escherichia coli*. *Appl. Microbiol. Biotechnol.* 100 9907–9916. 10.1007/s00253-016-7715-1 27401924

[B42] Wu H. Fan Z. Jiang X. Chen J. Chen G. Q. (2016b). Enhanced production of polyhydroxybutyrate by multiple dividing *E. coli*. *Microb. Cell Fact* 15:128. 10.1186/s12934-016-0531-6 27465264 PMC4964105

[B43] Xu X. Xian M. Liu H. (2017). Efficient conversion of acetate into phloroglucinol by recombinant *Escherichia coli*. *RSC Adv.* 7 50942–50948. 10.1039/c7ra09519h

[B44] Xu X. Xie M. Zhao Q. Xian M. Liu H. (2018). Microbial production of mevalonate by recombinant *Escherichia coli* using acetic acid as a carbon source. *Bioengineered* 9 116–123. 10.1080/21655979.2017.1323592 28574746 PMC5972924

[B45] Yuzawa S. Kim W. Katz L. Keasling J. D. (2012). Heterologous production of polyketides by modular type I polyketide synthases in *Escherichia coli*. *Curr. Opin. Biotechnol.* 23 727–735. 10.1016/j.copbio.2011.12.029 22244790

[B46] Zhang J. Gao X. Hong P. H. Li Z. J. Tan T. W. (2015). Enhanced production of poly-3-hydroxybutyrate by *Escherichia coli* over-expressing multiple copies of Narticle-title>Enhanced production of poly-3-hydroxybutyrate by *Escherichia coli* over-expressing multiple copies of NAD kinase integrated in the host genome. *Biotechnol. Lett.* 37 1273–1278. 10.1007/s10529-015-1797-1 25724717

[B47] Zhou Y. Qian B. (2010). Process technology of acetate acid and market analysis. *Chem. Ind.* 28 19–23.

