# Supplementary material for: Comparison of Glucose, Acetate and Ethanol as Carbon Resource for Production of Poly(3-Hydroxybutyrate) and Other Acetyl-CoA Derivatives
Source: Front Bioeng Biotechnol. 2020 Jul 23;8:833. doi: 10.3389/fbioe.2020.00833 (PMC7396591; doi:10.3389/fbioe.2020.00833)
Supplement: Supplementary file 1 [file Image_1.pdf]

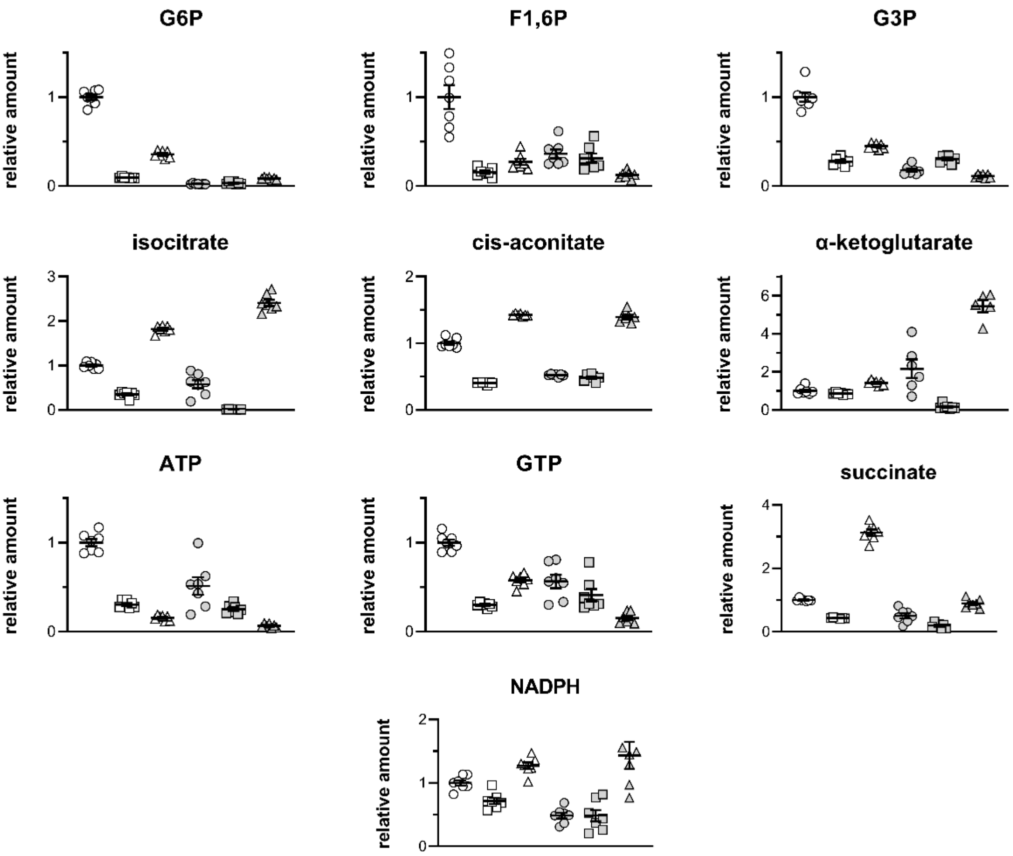

Fig S1 Relative quantification of metabolites in different strains at D1 and D2 stages in Fig 5B. Circle, Q3095 grown on glucose; square, Q3094 grown on ethanol; triangle, Q3140 grown on acetate; open symbol, D1 stage; filled symbol, D2 stage. G6P, glucose-6-phosphate; F1,6P, fructose 1,6-biphosphate; G3P, 3-phospho-glycerate. These experiments were carried out in septuplicate, and data represent mean ± SEM.
